# Awareness, perceptions and intent to comply with the prospective malaria vaccine in parts of South Eastern Nigeria

**DOI:** 10.1186/s12936-018-2335-0

**Published:** 2018-05-02

**Authors:** Uchechukwu M. Chukwuocha, Peter C. Okorie, Gregory N. Iwuoha, Sally N. Ibe, Ikechukwu N. Dozie, Bertram E. Nwoke

**Affiliations:** 1 Department of Public Health, Federal University of Technology, Owerri, Imo State Nigeria; 2 Department of Animal and Environmental Biology, Imo State University, Owerri, Imo State Nigeria

**Keywords:** Malaria, Vaccines, Awareness, Perception, Intent to comply

## Abstract

**Background:**

There are potentials of a malaria vaccine being developed sooner than expected. While focus is more on the development of a vaccine, less attention has been paid on the extent to which such vaccines could be well accepted and the readiness among caregivers to comply with its use in order to achieve the effectiveness of the vaccine in the malaria endemic areas. Compliance rates are influenced by the level of awareness, as well as the perception of the population. This cross-sectional study was aimed at assessing the awareness, perceptions and intent to comply with the prospective malaria vaccine by caregivers in Owerri West, South Eastern Nigeria.

**Methods:**

Structured pretested questionnaires were used to collect data from 500 randomly selected consenting care givers (mostly mothers). Items used to assess the intent to comply with the vaccine include willingness to accept and use the vaccine, and allow children to be vaccinated.

**Results:**

The study found that awareness of malaria as a public health problem was high (89.8%), but awareness about a prospective malaria vaccine was not high (48.2%). Up to 88.2% of respondents showed positive perception towards the vaccine, of which 65.2% had strong positive perception. The study found high level of intent to comply with the prospective malaria vaccine among the study group (95.6% positive). Significant association was established between caregivers perception and intent to comply with the prospective malaria vaccine (χ^2^ = 144.52; p < 0.0001).

**Conclusions:**

While malaria vaccine adoption is likely to be a welcome development in South Eastern Nigeria, proper consideration should be given to factors that are likely to influence people’s perceptions about vaccines in the plans/process of malaria vaccine development and vaccination programmes.

## Background

Malaria is a mosquito-borne disease which can be controlled in both humans and mosquitoes. Despite efforts and tools currently available for its control, malaria has remained the leading cause of morbidity and mortality in sub-Saharan Africa and other endemic countries [1, 2]. An obvious way of averting the spread of infectious diseases globally is the use of vaccines. The importance of the use of vaccines has successfully been demonstrated in the fight against many infectious diseases, such as polio, measles, diphtheria, tetanus, rabies and smallpox [3]. Therefore, the development and introduction of a malaria vaccine will represent an important achievement towards malaria elimination and eradication.

Malaria control strategies require a multifaceted approach, such as the use of insecticides, chemotherapy and development of affordable and efficacious malaria vaccines [4]. Significant advances have also been achieved in malaria control, including environmental approach and the introduction of artemisinin-based combination therapy [5, 6]. Progress has also been recorded on malaria vaccine development [7].

Malaria parasites do undergo morphological changes with variations in antigens as it advances from a sporozoite through the liver stage to the replicating cycle of the blood stage. It is possible for the parasite to evade protective immune responses of the host through a process of antigenic variation. This poses a huge challenge in malaria vaccine development especially when subjected to the common vaccines development pattern of weakening or killing the disease parasite. In consideration to the problems posed by this technique, the focus has shifted to identifying specific components or antigen of malaria parasite that can stimulate protection, aimed at providing effective immunity against malaria [8]. Significant successes for vaccination against malaria have been recorded on experimental studies in rodents, monkeys and human subjects in which attenuated sporozoites induced sterile protective immunity [7]. Hence, several malaria vaccines are currently in clinical trials and are expected to provide an improved strategy for malaria control [9]. Specifically, the RTS,S malaria vaccine candidate is undergoing clinical trials among children in three sub Saharan African countries [10, 11].

Making malaria vaccine available for routine use will be a major achievement, but the level of its acceptability, especially in the developing countries, could pose another considerable challenge that need to be addressed in order to achieve a successful implementation of the programme. Previous difficult experiences recorded in vaccination programmes in parts of sub-Saharan Africa, have raised concerns about the successful implementation of malaria vaccinations in endemic areas when developed [12]. For instance, the highest number of polio cases was once recorded in Nigeria, and that was blamed on low coverage and compliance of the vaccine in the country [13, 14].

While the focus is on malaria vaccine development, less attention has been paid on acceptance and readiness among caregivers to comply with malaria vaccinations. Compliance rates are influenced by the level of awareness as well as the perceptions of the population about the diseases in question [15]. Some cultural, social and religious circumstances in Nigeria have hindered previous vaccination programmes due to perceptions and beliefs in the causation of diseases and how they should be controlled [16–20].

Considering the potential influence of these factors on acceptance and compliance with the prospective malaria vaccine, this study investigated the awareness, perceptions and intent to comply with the prospective malaria vaccine by care givers in Owerri West, South Eastern Nigeria.

## Methods

### Study area

This study was carried out in Owerri West Local Government Area (LGA) of Imo State, South Eastern Nigeria. The area constitutes approximately a third of the capital city of Imo State, Nigeria. It has a total population of about 101,754 people as of 2006 census and covers an area of 297 km^2^. It is located in the tropical rain forest with climatic and environmental conditions that support malaria endemicity. The main language in the area is Igbo Language. Many also speak English and ‘Pidgin English’. The people are predominantly farmers, traders, civil servants and artisans. The vaccination rates for routine EPI in the area is 62.4% as at the last national demographic and health survey [21].

### Study design and sampling

The study employed a cross sectional descriptive design with the study population comprising of caregivers (mothers, in particular) resident within Owerri West LGA Nigeria. A pretested questionnaire was used to assess the awareness, perception and intent to comply with the prospective malaria vaccine. The questionnaire was validated using face and content validation. Thirty-five questionnaires were pretested in another community in Owerri West LGA with similar characteristics but not included for the actual study. The questionnaire was tested for reliability using Cronbach Alpha test [22] and a reliability coefficient of 0.71 was obtained.

Owerri West LGA has 16 communities grouped into for electoral divisions. A multi-stage technique was used in sampling of households for the study from the divisions. At the first stage, one community was randomly selected from each division through balloting. Systematic random sampling was then used in the selection of 500 households from which the study participants were drawn. Sampling started from the community centre of each community and households were selected at intervals of two households. This process went round the community until the required sample size for each selected community was reached. Additionally, at occasion non-household eligibility, the next household was selected. The next stage was the selection of eligible study participants from the households. The eligible participants were those who were resident in the area for the past 1 year, above 18 years of age and had children in their household.

Prior to data collection, the members of the selected communities were gathered at each community center for a sensitization exercise concerning the survey to be performed at their households and the need for their support. The appointment to that effect was scheduled in agreement with the community leaders who also helped to mobilize the members of their respective communities.

### Data collection

Data collection processes lasted for 4 months. Data was collected by administering structured pretested questionnaires to the study participants by members of the study group. For the selected participants, the study was once more introduced and informed consent was sought for their participation in the study. For those who gave their consent, the questionnaire was then elicited in the local (Igbo) language.

### Data analysis

The method of data analysis was descriptive. Data collected were presented in tables of frequency distribution and were all expressed as the percentage of the distribution. Responses on intent to comply with malaria vaccine were summarized as positive and negative intent by taking the average of the assessment items. Chi square was used to test for association between the level of perception and the intent to comply with prospective malaria vaccine at 5% significant level. Data analysis was performed on IBM-SPSS Statistics version 23.

## Results

### Socio-demographic characteristics

A total of 500 caregivers were involved in the study. The mean age and the standard deviations were 19.53 ± 7.24 years. Thirty percent of the respondents were between 41 and 50 years old. The majority (76%) were married and a greater proportion (37.6%) had tertiary education. They were predominantly Christians (99%). Over one-third of the respondents were farmers (36.4%), their family income structure was such that 20.4% earned between ₦38,000 and ₦47,000 and another 20.0% earned between ₦48,000 and ₦57,000; with 10% only earning above ₦57,000 per month (Table 1).

**Table 1:** Socio-demographic characteristics of respondents

Variables	Frequency (n = 500)	Percentage
Age (mean ± std. dev = 19.53 ± 7.24)		
Less than 20 years	50	10.0
21–30 years	130	26.0
31–40 years	100	20.0
41–50 years	150	30.0
51 years and above	70	14.0
Marital status		
Married	380	76.0
Divorced/separated	40	08.0
Widowed	80	16.0
Educational attainment		
Non-formal education	100	20.0
Primary	100	20.0
Secondary	112	22.4
Tertiary	188	37.6
Religion		
Christianity	498	99.6
Islam	2	0.40
African traditional religion	8	0.60
Occupation		
Farming	182	36.4
Business	168	33.6
Civil servants	90	18.0
Student	60	12.0
Family monthly income in Naira(₦)^a^		
Above 57,000	52	10.4
48,000–57,000	100	20.0
38,000–47,000	102	20.4
28,000–37,000	98	19.6
18,000–27,000	80	16.0
Less than 18,000	68	13.6

One Naira(₦) is equivalent to 345 USD

^a^ The earning cut offs were selected using Nigerian minimum wage of 18,000 naira. The intervals were then categorized based on the different earning categories at the local government level

### Awareness about malaria and the prospective malaria vaccine

**Fig. 1:**
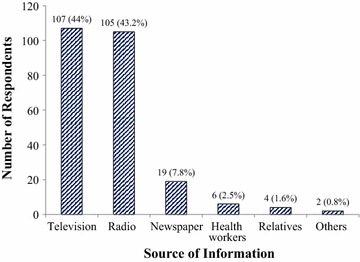
Primary source of information about malaria vaccine

All the respondents (100%) were aware of malaria, locally known as “Iba” (Table 2). The majority (89.8%) identified mosquito as the possible cause of malaria. Signs and symptoms of malaria were identified as fever (86.2%), headache (94%), cold (14.8%), swollen eyes (1%), bitter tongue (35.4%), deep yellow urine (26.4%). About half of the respondents (51.4%) were not aware of any prospective malaria vaccine. Among those who showed awareness about prospective malaria vaccine, 44 and 43.2% responded that media television and radio were their primary source of information (Fig. 1).

**Table 2:** Awareness of malaria and the prospective malaria vaccine, n = 500 (100%)

Variables	Frequency	Percentage
Awareness of the disease called malaria		
Yes	500	100.0
No	0	0.0
Local name of malaria		
Iba	350	70.0
Akum	130	26.0
Others	20	4.0
Awareness of causes of malaria		
Curse from God	31	6.2
Evil spirit	20	4.0
Mosquito	449	89.8
Manifestations of malaria experienced (symptoms)^a^		
Fever	431	86.2
Headache	470	94.0
Cold	74	14.8
Swollen eyes	5	1.0
Bitter tongue	177	35.4
Deep yellow urine	132	26.4
Running stomach	0	0
Awareness of prospective malaria vaccine		
No	257	51.4
Yes	243	48.6

^a^ Based on multiple response

### Perception about malaria vaccine

Majority of the respondents (60.2%) strongly agreed that malaria vaccine would prevent malaria. About 61.4% strongly agreed that everyone should receive malaria vaccine. Most of them (80.0%) also strongly agreed that they would take the malaria vaccine when produced, 86.6% strongly agreed the vaccine is not against their cultural belief. About 56.2% strongly agreed that malaria vaccine will save money spent on treatment while 41.8% strongly agreed that the vaccine will not have adverse health effects (Table 3).

**Table 3:** Perceptions about malaria vaccine, n = 500 (100%)

Variables	Frequency	Percentage
Malaria vaccine will prevent malaria		
Indifferent	40	8.0
Agree	159	31.8
Strongly agree	301	60.2
Everyone should receive malaria vaccine		
Disagree	15	3.0
Indifferent	75	15.0
Agree	103	20.6
Strongly agree	307	61.4
I will take the malaria vaccine when produced		
Indifferent	30	6.0
Agree	70	14.0
Strongly agree	400	80.0
Malaria vaccine is not against our cultural belief		
Indifferent	35	7.0
Agree	32	6.4
Strongly agree	433	86.6
Malaria is a major health problem requiring vaccine		
Disagree	30	6.0
Agree	167	33.4
Strongly agree	303	60.6
Malaria vaccine will save money spent on treatment		
Disagree	23	4.6
Indifferent	67	13.4
Agree	129	25.8
Strongly agree	281	56.2
Malaria vaccine will not have adverse health effect		
Strong degree	61	12.2
Disagree	109	21.8
Indifferent	20	4.0
Agree	101	20.2
Strongly agree	209	41.8
Malaria vaccine will save man hour lost to malaria		
Agree	199	39.8
Strongly agree	301	60.2

The summary of the perception on malaria vaccine was such that high level of perception was obtained among caregivers studied. Up to 88.2% showed positive perception about the vaccine, of which 65.2% showed strong positive perception (Table 4).

**Table 4:** Summary of perception on malaria vaccine among caregivers

Class	No of respondents	Percent
Negative perception	59	11.8
Positive perception (weak)	115	23.0
Positive perception (strong)	326	65.2
Total	500	100

### Intent to comply with the prospective malaria vaccine

Most of the respondents (96.2%) were ready to accept malaria vaccine, if eventually developed and deployed. Similar number of study participants (94.2%) responded that they will use the malaria vaccine when produced, and 96.2% indicated that they will allow their children to be immunized with the malaria vaccine (Table 5). Summarily, 95.6% of the study group showed positive intent to comply with the prospective malaria vaccine against 4.4% that were negative to it.

**Table 5:** Intent to comply with the prospective malaria vaccine

Variables	Frequency	% (n = 500)
Level of acceptance of prospective malaria vaccine		
Will accept	481	96.2
Will not accept	11	2.2
No response	8	1.6
Will use malaria vaccine when produced		
Yes	471	94.2
No	29	5.8
Will allow children to be immunized with malaria vaccine		
Yes	481	96.2
No	19	3.8

### Perception towards prospective malaria vaccine and intent to comply

Among the care givers that showed negative perception about the malaria vaccine, 37.3% were of negative intent to comply with the vaccine compared to 2.6% found among those with positive perception. All the care givers that showed strong positive perception equally showed positive intent to comply (Table 6). Significant association was found between caregivers perception and intent to comply with prospective malaria vaccine (χ^2^ = 144.52; p < 0.0001).

**Table 6:** Perception towards prospective malaria vaccine and intent to comply

Perception	Negative intent	Positive intent	Total
Negative perception	19 (37.3%)	32 (62.7%)	51
Positive perception (weak)	3 (2.6%)	112 (97.4%)	59
Positive perception (strong)	0 (0.0%)	326 (100)	115
Total	22 (4.4%)	478 (95.6%)	326

Statistical test: χ^2^ = 144.52; p < 0.0001

### Willingness to pay for malaria vaccine and reasons

The respondents who would be willing to pay for the vaccine are only 40.6%, of which 64% indicated that they will be willing to do so to enable the vaccine to be readily available. On the other hand, among those who may not be willing to pay for the vaccine, the majority (45.1%) were of the view that the cost of the vaccine is not likely to be affordable by households, 25% responded that the vaccine will not be accessible while 20.9% stated that it may have adverse effect on children (Table 7).

**Table 7:** Willingness to pay for malaria vaccine and reasons

Response	Frequency	Percentage (%)
Willing to pay for malaria vaccine		
Yes	203	40.6
No	297	59.4
Total	500	100
Reason for being willing to pay for malaria vaccine		
For continuous production	130	64.0
To promote total acceptance	73	36.0
Total	203	100
Reason for not being willing to pay for malaria vaccine		
Cost not affordable by households	134	45.1
It will not be accessible	75	25.3
Fear of adverse effect on children	62	20.9
Child’s father won’t support vaccination	23	7.7
Against culture	2	0.7
Against religion	1	0.3
Total	297	100

## Discussion

Making malaria vaccine available for routine use will be a major hallmark for the control and eventual elimination of malaria. However, the extent of acceptance and compliance with this vaccine will depend on awareness and general perceptions about malaria itself and the potency of a vaccine in its control and elimination.

In the present study, most of the respondents are very much aware of the cause and dynamics of malaria. The degree of awareness about malaria recorded in this study could possibly be as a result of high level of education of the caregivers in this study. Educational attainment has been previously shown to be associated with the awareness of malaria and its dynamics in parts of sub-Saharan Africa [23–26]. Other factors that tend to influence awareness towards malaria and a possible malaria vaccine include religion, house-hold income and marital status [27].

This study found that awareness about a prospective malaria vaccine is low among the caregivers studied. This indicates that it may take more than just formal education attainment for people to have the necessary awareness needed to implement health interventions such as the malaria vaccine. Two studies, one in Kenya [28] and the other in Ghana [29], found that consistent communication and enlightenment of the populace on contemporary public health issues tend to have played a major role. There needs to be consistent information, education and communication on public health issues especially new and prospective interventions.

The respondents acknowledged that a malaria vaccine could be a very effective preventive tool against malaria disease. They are also very willing to accept and use the malaria vaccine and have their children vaccinated against malaria if the vaccine is fully developed and made available. Such encouraging reactions on malaria vaccine could be due to positive results achieved from previous vaccination programmes [29].

However, the caregivers were skeptical on the affordability of the vaccines, if households are required to pay for it. Cost and affordability have been associated with non-compliance of some health interventions especially in low resource areas and therefore led to the limited successes achieved in the control of diseases such as malaria [5]. On the contrary interventions that are associated with no cost to the people have achieved better results. This factor should, therefore, be put into prior considerations during planning and implementation of malaria vaccination programmes. Attention should also be paid towards winning the support of the heads of households since they usually pay for the cost of family health needs. A study in Nigeria showed that majority of people who reported that they will not go for vaccination indicated that their reason was that their husbands will not support it [30]. Furthermore, the awareness of any potential adverse effects should be part of community education about the vaccine as this would enhance acceptability and compliance.

It suffices to know among factors such as belief, cultural practice and efficacy, side effect could be inevitable factors influencing acceptance of and compliance with the malaria vaccine [30, 31]. Hence the acceptability of malaria vaccine may be dependent on the level of side effects of the vaccines when eventually developed and introduced.

Intent to comply with the prospective malaria vaccine was significantly associated with caregivers perception, hence this study suggests that consideration should always be given to factors that are likely to lower people’s perception about vaccines in the development of new vaccines.

This study recorded some limitations. First, the study population was highly literate therefore the findings of this study may not represent similar outcomes is less literate populations. Second, the content of the study questionnaire instrument was initially quite large reflecting the large scope of the study. This led to redundancy on the side of the respondents to respond to all the question; as a result, the study was eventually divided into two phases.

## Conclusions

Many of the care givers in South Eastern Nigeria are willing to accept and use the malaria vaccine when developed. This is very encouraging for the malaria vaccine implementation programme in the area. Such factors as high literacy level and awareness about malaria could have contributed to this situation. Similar studies should be carried out in other malaria endemic area to assess their level of preparedness for the eventual implementation of the malaria vaccination programmes. Consideration should also be given to factors that are likely to influence people’s perceptions about vaccines during development and planning for vaccination programme implementation. There is the need for expanded and continuous public health information, education and communication particularly on contemporary health issues such as malaria and vaccinations. This will enable easier implementation as well as more acceptance and compliance towards the sustainable control and eventual elimination of malaria.
